# Circulating Exosomal MiR-107 Restrains Tumorigenesis in Diffuse Large B-Cell Lymphoma by Targeting 14-3-3η

**DOI:** 10.3389/fcell.2021.667800

**Published:** 2021-04-27

**Authors:** Jiarui Liu, Yang Han, Shunfeng Hu, Yiqing Cai, Juan Yang, Shuai Ren, Yi Zhao, Tiange Lu, Xiangxiang Zhou, Xin Wang

**Affiliations:** ^1^Department of Hematology, Shandong Provincial Hospital, Cheeloo College of Medicine, Shandong University, Jinan, China; ^2^Department of Hematology, Shandong Provincial Hospital Affiliated to Shandong First Medical University, Jinan, China; ^3^School of Medicine, Shandong University, Jinan, China; ^4^Shandong Provincial Engineering Research Center of Lymphoma, Jinan, China; ^5^Branch of National Clinical Research Center for Hematologic Diseases, Jinan, China; ^6^National Clinical Research Center for Hematologic Diseases, The First Affiliated Hospital of Soochow University, Suzhou, China

**Keywords:** diffuse large B-cell lymphoma, microRNAs, miR-107, exosome, 14-3-3η

## Abstract

Exosomes, nanometer-sized membranous vesicles in body fluids, have emerged as promising non-invasive biomarkers for cancer diagnosis. However, the function of exosomes in diffuse large B-cell lymphoma (DLBCL) remains elusive. This study aimed to investigate the role of exosomal miR-107 in lymphomagenesis and explore its clinical significance. In this study, decreased exosomal miR-107, miR-375-3p, and upregulated exosomal miR-485-3p were detected in the plasma of DLBCL patients and showed potential diagnostic value. Downregulated miR-107 expression was associated with advanced Ann Arbor stage, high IPI score, LDH, and β_2_-MG level in DLBCL patients. Overexpression of miR-107 by miR-107 Agomir significantly abrogated cell proliferation, induced apoptosis, and inhibited cell invasion *in vitro*, and repressed tumor growth *in vivo*. Moreover, the downregulation of miR-107 went in the opposite direction. The target genes of miR-107 were mainly enriched in the PI3K-Akt, Hippo, and AMPK signaling pathways. Notably, upregulated 14-3-3η (YWHAH) was suppressed by miR-107 in DLBCL, suggesting that miR-107 may restrain tumorigenesis by targeting 14-3-3η. In summary, this study unveils the function of miR-107 in lymphomagenesis, highlighting its potential as a diagnostic and prognostic indicator and as a new therapeutic target in the management of DLBCL.

## Introduction

Diffuse large B-cell lymphoma (DLBCL) represents the most common subtype of non-Hodgkin lymphoma (NHL) and accounts for 30–40% of all newly diagnosed cases. Approximately 75% of DLBCL patients following standard rituximab plus cyclophosphamide, doxorubicin, vincristine, and prednisone (R-CHOP) treatment reach complete remission (Wei Y.H. et al., 2020); however, 30–40% present relapsed or refractory to R-CHOP treatment within a year after diagnosis (Maurer et al., 2016). These groups of patients usually suffer an unfavorable outcome, with a 1-year overall survival (OS) of less than 20% (Vitolo et al., 2017). Approaches targeting the different mechanisms of the pathogenesis of DLBCL may provide a strategy for increasing the therapeutic armamentarium. Early identification of high-risk patients may allow for active treatment strategies to be considered.

Until recently, the diagnosis of DLBCL still relies on tissue specimen examination, which is invasive and expensive. International prognostic index (IPI) score (based on age, tumor stage, LDH, performance status, and the number of extranodal disease sites), gene expression profiling, and immunohistochemical analysis are usually used to predict survival time. Given the heterogeneity of DLBCL, patients with identical IPI scores may exhibit striking heterogeneity in outcomes (Voltin et al., 2020). Moreover, IPI also shows limitations in estimating treatment efficacy. Accurate prognostic evaluation methods are needed to overcome the limitations of traditional methods.

Exosomes, small (30–150 nm) bilayer membrane vesicles, are considered as cargo to transfer material (proteins, nucleic acids, lipids, metabolites, and organelles) from parental cells to targeted cells via ligand–receptor interactions. Exosomes with their biologic cargo were reported to play a pivotal role in mediating the immune response, tumor metastasis, angiogenesis, bone marrow reeducation, and drug resistance (Liu and Wang, 2019; Daassi et al., 2020; Longjohn et al., 2021). Accumulating evidence proves the potential of exosomes to serve as candidate biomarkers, therapeutic targets, drug delivery vehicles, or vaccines in a range of diseases (Sinha et al., 2021).

Due to the characteristics of being highly informative, stable, and present in all kinds of body fluids, exosomal miRNAs represent optimal biomarkers over traditional miRNAs for diagnosis, prognosis, and predicting therapy response (Zhou et al., 2020b; Yu et al., 2021). For example, miRNAs encapsulated within exosomes are protected from RNase in biological liquids. Besides, miRNA profiles encapsulated into exosomes are prespecified, which refers to the fact that exosomal miRNAs reflect specific information of parental cells. In line with this, accumulating evidence proves exosomal miRNAs as novel biomarkers in early detection, serial monitoring, and prognosis evaluation of hematological malignancies (Drees and Pegtel, 2020; Gargiulo et al., 2020; Seimiya et al., 2020). However, until recently, there are still not abundant studies regarding the predictive role of exosomal miRNAs in DLBCL.

The findings of this study provide evidence of the diagnostic value of circulating exosomal miRNAs (miR-107, miR-375-3p, and miR-485-3p) in patients with DLBCL. Exosomal miR-107 shows a close relationship with advanced clinicopathological factors. Furthermore, miR-107 has been demonstrated to suppress cell proliferation and invasion in DLBCL cells by targeting 14-3-3η. This study provides a novel therapeutic option to improve the prognosis of DLBCL and promote the clinical application of exosomes.

## Materials and Methods

### Patients and Cell Lines

A total of 42 plasma samples were collected from DLBCL patients diagnosed from 2017 to 2019 at Shandong Provincial Hospital. Plasma from 31 volunteers were randomly collected at the same period as controls. Informed consent was obtained from each patient. Peripheral blood mononuclear cells (PBMCs) were isolated by the Ficoll-Hypaque density gradient centrifugation method (TBD Science, Tianjin, China). The study was abided by Helsinki protocol and approved by the Medical Ethical Committee of Shandong Provincial Hospital.

DLBCL cell lines OCI-LY1, OCI-LY3, OCI-LY8, and OCI-LY10 were purchased from American Type Culture Collection (ATCC, Manassas, VA, United States). Cells were cultured in IMDM with 10% fetal bovine serum (FBS, GIBCO, MD, United States) and 1% double antibiotics, incubated at 37°C in 5% CO_2_. All cells were examined for mycoplasma infection periodically.

### Cell Transfection

MiR-107 Agomir/Antagomir control or miR-107 Agomir/Antagomir were purchased from RIBOBIO (Shenzhen, China) and transfected into DLBCL cell lines using the ribo*FECT*^TM^ CP Transfection Kit according to the manufacturer’s instructions.

### Exosomes Isolation and Identification

Plasma was treated at 2,000 g for 20 min before storage at −80°C for exosome isolation. Plasma exosomes were extracted by the exoEasy Maxi kit (Umibo, Shanghai, China). Exosome isolation was performed in accordance with the manufacturer’s instructions and detected by transmission electron microscopy (TEM) and Western blot analysis.

### RNA Extraction and Quantitative Real-Time PCR

Total RNAs were isolated from exosomes using TRIzol reagent. Complementary DNA (cDNA) was amplified using the miRNA 1st strand cDNA synthesis kit (MR101-02, Vazyme, Nanjing, China). Quantitative real-time PCR (qRT-PCR) reactions with cDNA as a template were conducted on LightCycler480 II (Roche, Basel, Switzerland) using a SYBR Green PCR kit (MQ101-02, Vazyme). Quantification was normalized by U6 and calculated using the 2^–Δ^ ^Δ^ ^*Ct*^ method (Yang et al., 2020). The primer sequences are shown below.

miR-107: 5′GTCGTATCCAGTGCAGGGTCCGAGGTATT CGCACTGGATACGACTGATAG3′ and 5′GCGAGCAGCAT TGTACAGGG3′;

miR-375-3p: 5′GTCGTATCCAGTGCAGGGTCCGAGGTA TTCGCACTGGATACGACTCACGC3′ and 5′GCGTTTGT TCGTTCGGCTC3′;

miR-485-3p: 5′CGCTTCACGAATTTGCGTGTCAT3′ and 5′CGCGTTCAACGGGTATTTAT3′

### Protein Extraction and Western Blot Analysis

Cell lysate extraction, total protein concentration determination, and gel electrophoresis were exerted according to the previous study (Zhou et al., 2017). Primary antibodies were Tsg101 (ab125011, Abcam, Cambridge, United Kingdom), CD9 (ab92726, Abcam), 14-3-3η (15222-1-AP, proteintech, Wuhan, China).

### Cell Proliferation, Apoptosis and Invasion Assays

The CCK-8 assay kit (Dojindo, Tokyo, Japan) was used to assess cell proliferation. Cell apoptosis was detected by the Annexin V-PE/7AAD apoptosis detection kit (BD Biosciences, Bedford, United States) followed by the procedure. Doxorubicin (0.5 μM), a well-known apoptosis inducer and conventional chemotherapy in DLBCL, was used to treat with miR-107 overexpressed cells for 12 h and then cell apoptosis was detected. The transwell assay was applied to evaluate cell invasion ability. The upper chamber (8 μm, Corning, Tewksbury, United States) was coated with Matrigel (1:3 dilution, BD Biosciences, San Jose, United States), and 200 μl serum-free medium with 2 × 10^5^ transfected cells were seeded into the upper chamber and 600 μl medium containing 10% FBS was placed in the lower chamber. After incubation at 37°C for 48 h, cells in the upper chamber were removed, and the membrane was fixed with iced methanol and stained with 0.1% crystal violet. The above protocols were performed according to the previous study (Yang et al., 2020).

### Immunofluorescence

Slides were deparaffinized with xylene, rehydrated, and unmasked following standard immunohistochemical methods (Zhou et al., 2020a). The slides were first incubated with primary antibody anti-14-3-3η (ab191060, Abcam) at 1:200 at 4°C overnight. Then, the slides were incubated with Invitrogen Alexa Fluor Plus 594 goat anti-rabbit IgG secondary antibody (1:500, A32740, Invitrogen). Immunofluorescence signals were detected under LEICA TCS SP8.

### Dual Luciferase Reporter Activity Assay

The 3’-UTR of 14-3-3η, containing the binding site of miR-107 and corresponding mutant sequences, was constructed into the pmirGLO vector and was cotransfected with miR-107 Agomir into HEK293T cells for 48 h. Then the dual luciferase reporter assay was performed according to instructions of the Dual-Luciferase Reporter Assay system (Promega, Madison, United States). Firefly luciferase activities were normalized by Renilla activities.

### Mouse Xenograft Model

Ten severe combined immunodeficiency (SCID) beige female mice, 3–4 weeks old, were purchased from Charles River (Beijing, China). Experiments were abided by ARRIVE guidelines and approval of the Animal Care and Use Committee of Shandong Provincial Hospital (NO. 2020 [768]). After 1 week of preadaptation, they were inoculated subcutaneously in right back of OCI-LY8 cells transfected with either Agomir NC or miR-107 (1 × 10^7^ cells suspended in 100 μl PBS and mixed with 100 μl matrigel). Mice were sacrificed on day 28 and the tumor volume was calculated as V = length (cm) × width (cm)^2^/2 (Zhou et al., 2020a).

### Identification of Differentially Expressed MiRNAs

MiRNA array expression profile data GSE117063 and GSE29493 were downloaded from the Gene Expression Omnibus (GEO) database^1^. Differentially expressed miRNAs (DEMs) were analyzed using GEO2R with the following criteria: *p* < 0.05 and |logFC| ≥ 2. The expression of 100 DEMs was imported into Morpheus, an online heat map–making tool. Coloring illustrates the high expression (red) and low expression (blue) of miRNA.

### Target Genes of MiR-107 and Enrichment Analysis

TargetScan^2^, miRDB^3^, miRTarbase^4^, PicTar^5^, and miRWalk2.0^6^ were used to predict the target genes of miR-107. A Venn diagram shows the intersections of four groups of target genes. The lncRNA–mRNA network was predicted using DIANA-LncBase v.2. We further validated the expression of lncRNAs in the GEO dataset (GSE97336) with the criteria: *p* < 0.05 and |logFC| ≥ 2. Gene ontology (GO) and Kyoto Encyclopedia of Genes and Genomes (KEGG) enrichment analyses were performed using DAVID online tool^7^. The cluster of genes was shown in a pathway annotation network in the plugin ClueGO (2.5.4) of Cytoscape (3.7.2).

### Statistical Analysis

The statistical significance between the two experimental groups was assessed by a two-tailed Student *t-*test. A one-way ANOVA-Dunnett test was used for comparing more than two groups. Correlations were estimated by the Pearson correlation coefficient. Kaplan–Meier survival analysis was performed to estimate progression-free survival (PFS) and analyzed by log-rank test. By analyzing the receiver operating characteristic (ROC) curve of exosomal miRNAs, the optimal cutoff point was calculated to distinguish the DLBCL patients from the healthy controls. All experiments were performed in triplicate and repeated three times. Graph drawing and statistical analysis were performed with GraphPad Prism v.8.0. Values were expressed as mean ± standard deviation (s.d.). *p* < 0.05 was considered statistically significant.

## Results

### Identification of DEMs in DLBCL

**FIGURE 1:**
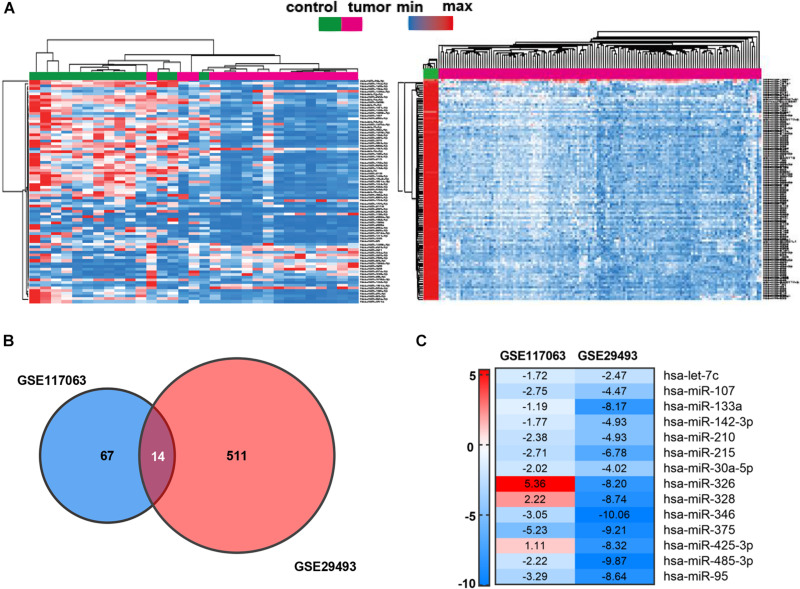
Identification of DEMs in DLBCL. **(A)** Heat map of the top 100 DEMs of GSE117063 (left) and GSE29493 (right). The lateral axis represents the samples, and the longitudinal axis represents the DEMs. Green indicates control specimens, pink indicates DLBCL specimens. Red indicates upregulation, blue indicates downregulation. **(B)** Identification of 14 DEMs overlapping between both GEO datasets. | log_2_FC| ≥ 1 and *p*-value < 0.05 set as cutoff criteria. **(C)** Heat map of DEM expression in GSE117063 and GSE29493. Red indicates upregulation; green indicates downregulation. The value of each cell indicates the fold changes of miRNAs expression.

To identify the candidate miRNAs in the carcinogenesis and progression of DLBCL, we screened miRNA expression using two standardized GEO datasets (GES117063 and GSE29493). The heat map in Figure 1A presents the expression of the top 100 DEMs of the two datasets among DLBCL patients and healthy controls (twofold plus *p* < 0.05). In the Venn diagram, the overlap of the two datasets contains 14 DEMs (Figure 1B), and the expression patterns of the DEMs in the two GEO datasets are shown in the heat map (Figure 1C). After eliminating the miRNAs (miR-326, miR-328, and miR-425-3p) with inconsistent expression, the remaining 11 downregulated miRNAs (let-7c, miR-107, miR-133a, miR-142-3p, miR-210, miR-215, miR-30a-5p, miR-346, miR-375, miR-485-3p, miR-95) were selected for further investigation.

### Targets Prediction and Functional Enrichment Analysis of DEMs

**FIGURE 2:**
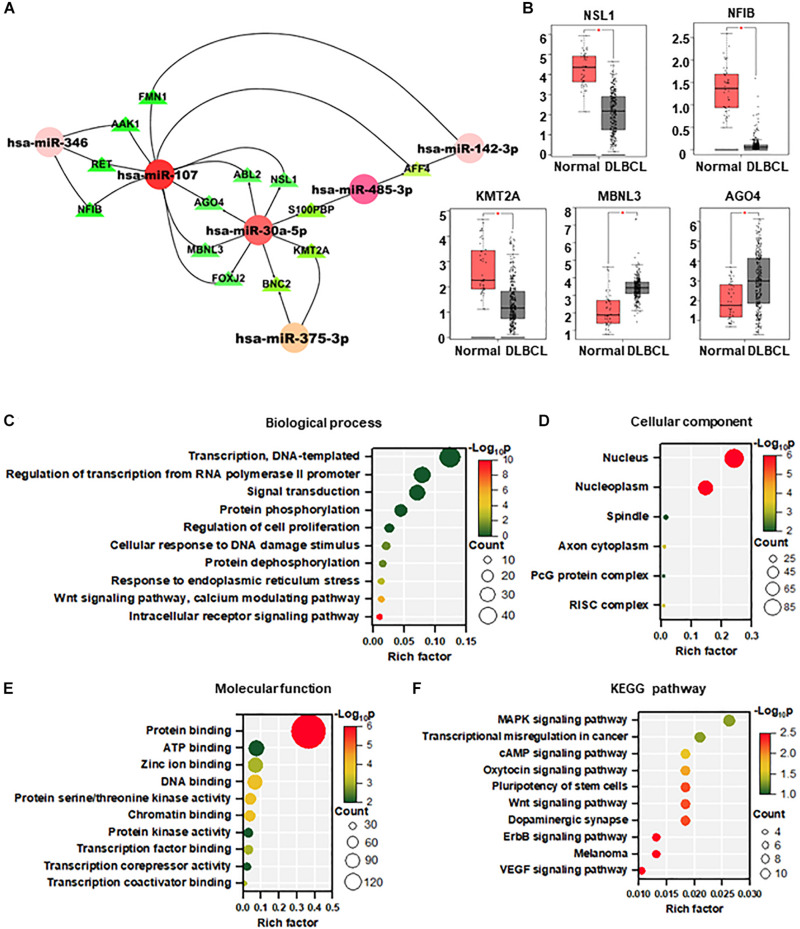
Targets prediction and functional enrichment analysis of DEMs. **(A)** MiRNA–mRNA interaction network based on the hot miRNAs. **(B)** Target gene expression in DLBCL from the GEPIA database (normal = 337, DLBCL = 47). **(C–F)** Visualization of GO enrichment analysis **(C–E)** and KEGG pathway enrichment analysis **(F)** of 484 target genes of DEMs. The *Y-*axis stands for terms, and the *X-*axis represents the rich factor. The significance of the term is described by the color with red representing the highest significance. The count of the gene enrichment in the term is described by the size of the node. GO, gene ontology; KEGG, Kyoto Encyclopedia of Genes and Genomes.

A total of 484 targeted genes of the 11 DEMs were predicted by the miRWalk 3.0 and miRDB database. The miRNA–mRNA interacting network based on the shared miRNAs was visualized using Cytoscape, which may reveal new interactions in the pathogenesis of DLBCL. As shown in Figure 2A, most of the target genes were associated with miR-107 and miR-30a-5p. We further analyzed the Gene Expression Profiling Interactive Analysis (GEPIA) database of DLBCL and found 5 upregulated genes (NSL1, NFIB, MBNL3, AGO4, KMT2A).

To analyze the biological function of DEMs in DLBCL, GO and KEGG pathway enrichment analysis were performed by DAVID. The top 10 most enriched categories in the GO and KEGG pathways were screened. In the biological process, these DEMs were mainly enriched in transcription, regulation of transcription from polymerase II promoter, and signal transduction (Figure 2B). In the cellular component, DEMs were mainly enriched in the nucleus, nucleoplasm, and spindle (Figure 2C). In terms of molecular function, DEMs were mainly enriched in protein binding, ATP binding, and zinc ion binding (Figure 2D). In the KEGG pathway enrichment analysis, DEMs were mainly enriched in MAPK, transcriptional misregulation in cancer, and cAMP signaling pathways (Figure 2E).

### Circulating Exosomal MiRNAs Exerted Diagnostic and Prognostic Value in DLBCL

**FIGURE 3:**
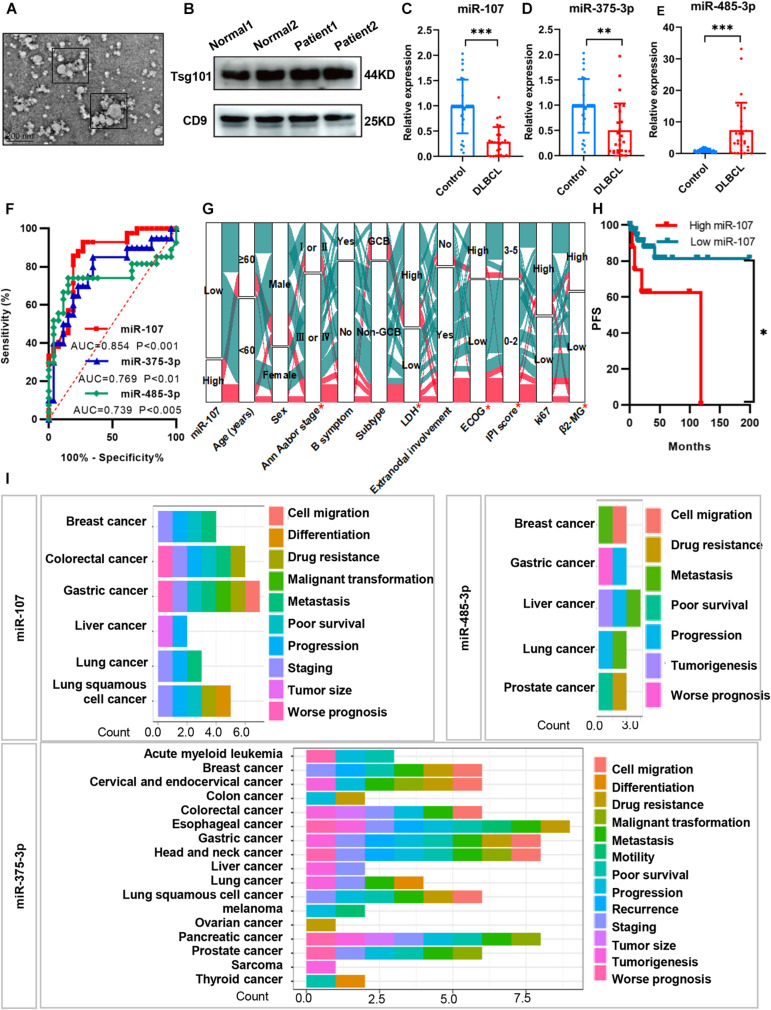
Circulating exosomal miRNAs exerted diagnostic and prognostic value in DLBCL. **(A)** TEM image of exosome particles shows typical cup-shaped morphology. **(B)** Western blot analysis of plasma exosomes with exosomal biomarker Tsg101 and CD9. **(C–E)** QRT-PCR analysis of the expression levels of exosomal miR-107, miR-375-3p, and miR-485-3p in the plasma of DLBCL (*n* = 42) and healthy controls (*n* = 31). **(F)** Receiver operating characteristic curves of biomarkers. **(G)** Alluvial diagram of miR-107 expression in groups with different clinical characteristics. Red represented high expression of miR-107; blue represented low expression of miR-107. **(H)** Kaplan–Meier analysis of DLBCL patients from Shandong Provincial Hospital. **(I)** Prognosis of miRNAs in various kinds of cancers from miRNAcancerMap database. Data are mean ± s.d., n ≥ 3. *p < 0.05, **p < 0.01, and ***p < 0.001. TEM, transmission electron microscopy. ROC, receiver operating characteristics.

To determine whether the DEM expression indeed reflects exosomal miRNAs in the plasma of DLBCL patients, we first isolated exosomes from 42 DLBCL patients and 31 healthy volunteers. Consistent with the previous description by Liu et al. (2020), the exosomes we isolated were approximately 30–150 nm in diameters, typical cup-shaped morphology, and expressed exosomal biomarkers Tsg101 and CD9 (Figures 3A,B). Based on the qRT-PCR analysis, we identified exosomal miR-107 (*p* < 0.001) and miR-375-3p (*p* < 0.01) downregulated, and miR-485-3p (*p* < 0.001) upregulated in the plasma of DLBCL patients (Figures 3C–E).

To test if the exosomal miRNAs were able to separate the DLBCL patients from the healthy people, we performed ROC curves and calculated the area under the curve (AUC). In all patients, miR-107, miR-375-3p, and miR-485-3p could significantly separate the groups. The AUC of miR-107, miR-375-3p, and miR-485-3p was 0.854, 0.769, and 0.739 with the best cutoff values of 0.67, 0.64, and 0.60, respectively (Figure 3F). The above results suggest that plasma exosomal miR-107, miR-375-3p, and miR-485-3p could be used as potential indicators in distinguishing DLBCL patients from healthy people.

We next identified whether exosomal miRNAs were of clinical prognostic significance in DLBCL patients. As shown in Figure 3G, compared with those with high miR-107 expression, patients with low expression of exosomal miR-107 showed more advanced Ann Arbor stage, higher levels of ECOG score, LDH, β_2_-MG, IPI score (all *ps* < 0.05), and shorter PFS (*p* < 0.05). But there were no significant differences between miR-107 expression and age, sex, B symptom, subtype, extranodal involvement, and Ki67 (all *ps* > 0.05). The prognostic significance of miR-107 was further confirmed in the database of miRCancerMap. As presented in Figure 3H, miR-107 is mainly associated with poor prognosis, progression, staging, and malignant transformation. Additionally, the functions of miR-375-3p are reported in many cancer types, which are related to tumorigenesis, staging, recurrence, metastasis, and so on. However, miR-485-3p has not been well illustrated, which mainly participated in cancer progression, metastasis, and drug resistance. Taken together, miR-107 shows strong prognostic relevance to DLBCL patients, but the biological mechanisms underlying this effect remain unclear.

### MiR-107 Regulated Proliferation, Apoptosis, and Invasion of DLBCL Cells

**FIGURE 4:**
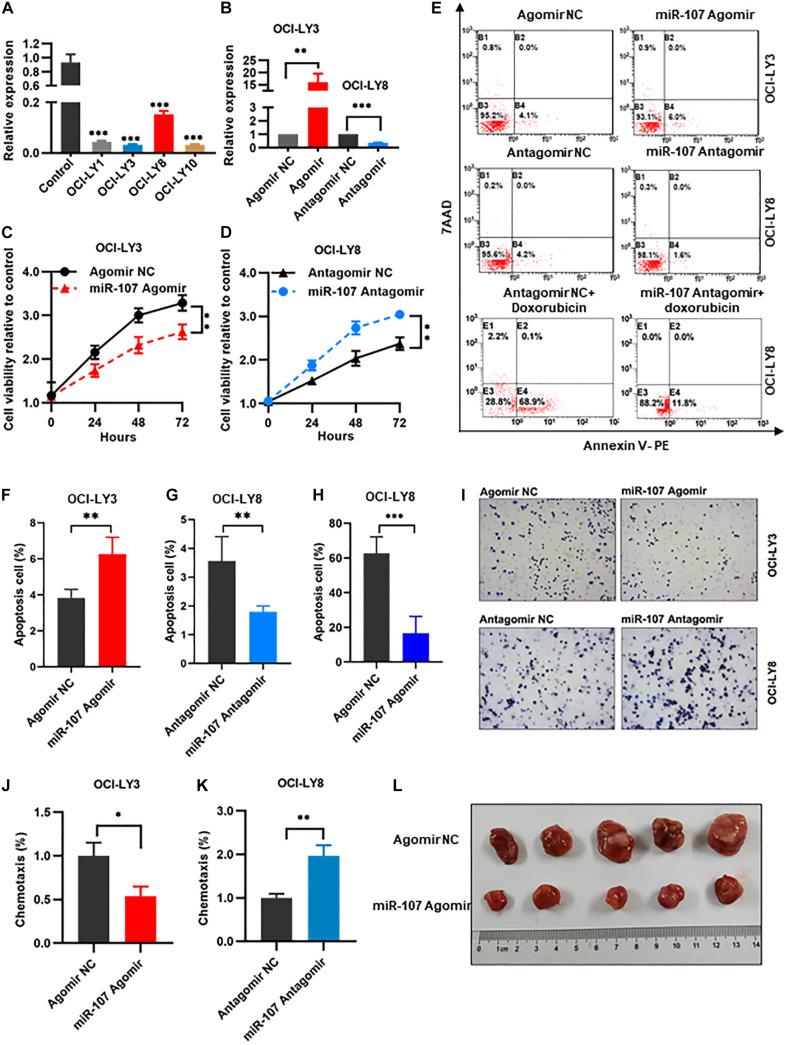
MiR-107 regulated proliferation, apoptosis, and invasion of DLBCL cells. **(A)** QRT-PCR analysis of miR-107 in DLBCL cell lines and controls. PBMCs of healthy donors were used as control. **(B)** QRT-PCR analysis of transfection efficiency in OCI-LY3 transfected with miR-107 Agomir and OCI-LY8 transfected with miR-107 Antagomir. **(C,D)** MiR-107 overexpression inhibited while miR-107 downregulation promoted cell proliferation of DLBCL cell lines. **(E–H)** MiR-107 overexpression promoted while miR-107 downregulation inhibited cell apoptosis of DLBCL cell lines **(E–G)**. Doxorubicin induced cell apoptosis, which could be reversed by miR-107 downregulation **(E,H)**. **(I–K)** MiR-107 overexpression was inhibited while miR-107 downregulation promoted cell invasion of DLBCL cell lines. **(L)** Representative images of tumors in OCI-LY8 xenograft mice transfected with miR-107 Agomir or Agomir NC. Data are mean ± s.d., n ≥ 3. *p < 0.05, **p < 0.01, and ***p < 0.001. PBMCs, peripheral blood mononuclear cells; TEM, transmission electron microscopy.

To explore the biological function of miR-107 in DLBCL pathogenesis, gain- and loss-of-function experiments were carried out. MiR-107 expression was significantly decreased in DLBCL cell lines (OCI-LY1, OCI-LY3, OCI-LY8, and OCI-LY10) compared with normal controls (*p* < 0.01, Figure 4A). OCI-LY3 cells with the lowest miR-107 expression were used to generate miR-107 overexpression cells by infection with miR-107 Agomir. Meanwhile, OCI-LY8 cells with the highest miR-107 expression was used to generate miR-107 knockdown cells by infection with miR-107 Antagomir (Figure 4B).

We evaluated the regulation role of miR-107 in cell proliferation by CCK8 assay in OCI-LY3 and OCI-LY8 cells. Compared with the Agomir NC group, overexpression of miR-107 significantly abrogated cell proliferative viability in OCI-LY3 cells (*p* < 0.01, Figure 4C). On the contrary, cell proliferative ability was significantly increased in OCI-LY8 cells transfected with miR-107 Antagomir (*p* < 0.01, Figure 4D). Furthermore, compared with scramble controls, the overexpression of miR-107 promoted cell apoptosis in OCI-LY3 cells (4.1 vs. 6.0%, *p* < 0.01) while downregulating miR-107 went in the opposite direction (4.2 vs. 1.8%, *p* < 0.01, Figures 4E–G). Doxorubicin is one of the commonly used cytotoxic drugs in the chemotherapy of DLBCL, which was reported to suppress lymphoma growth by inducting caspase 3-dependent apoptosis (Tayeh and Ofir, 2018). In this study, we use doxorubicin (0.5 μM) to treat miR-107 overexpressed cells. As shown in the figure, doxorubicin induced OCI-LY8 cell apoptosis, and suppression of miR-107 expression markedly potentiated doxorubicin-induced apoptosis (68.9 vs. 11.8%, Figures 4E,H). We next explored the role of miR-107 in cell invasion by transwell assay. MiR-107 upregulation weakened the invasive potential of OCI-LY3 cells (*p* < 0.05), and downregulating of miR-107 significantly stimulated the invasion ability of OCI-LY8 cells (*p* < 0.01, Figures 4I–K).

To further confirm the tumor suppressor role of miR-107 *in vivo*, of note, OCI-LY8 cells transfected with miR-107 Agomir or Agomir NC were inoculated subcutaneously in the right back of SCID beige mice. Consistent with the aforementioned results, the tumor volume of OCI-LY8 xenograft mice transfected with miR-107 Agomir were significantly smaller than those of the control group (Figure 4L). Collectively, the above results demonstrate that miR-107 represses cell proliferation and invasion *in vitro* and tumor growth *in vivo*.

### Enrichment Analysis and Regulatory Networks of miR-107

**FIGURE 5:**
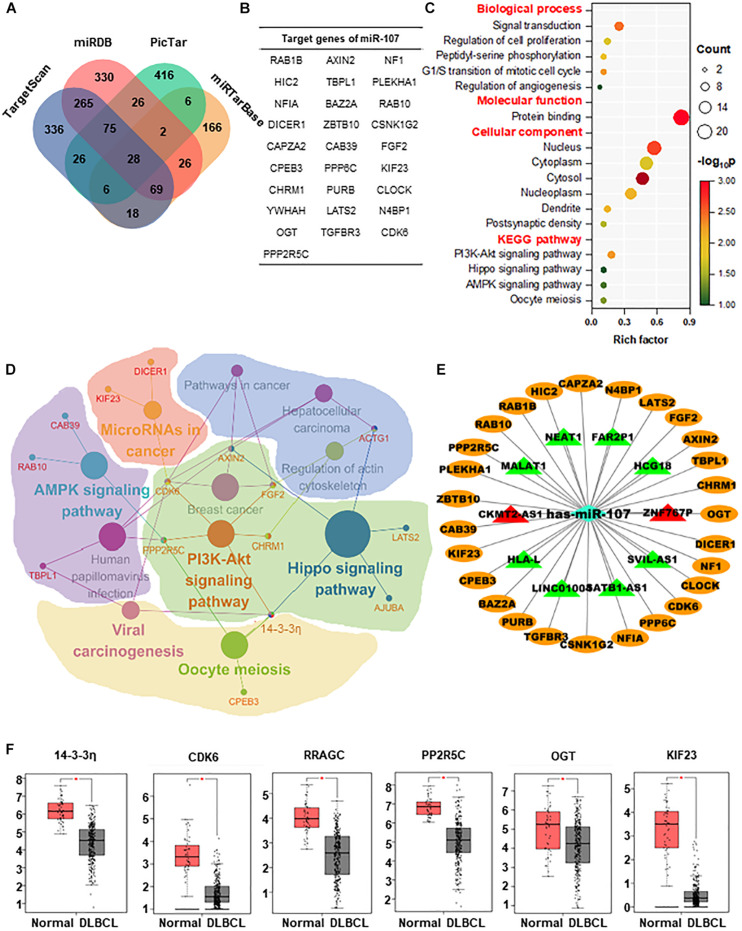
Enrichment analysis and regulatory networks of miR-107. **(A)** Venn diagram indicates 28 potential target genes of miR-107 predicted by four databases, including TargetScan, miRDB, miRTarBase, and PicTar. **(B)** Table of target genes of miR-107. **(C)** GO enrichment and KEGG analysis of target genes by DAVID. **(D)** Representative biomolecular network about 28 genes targeted by miR-107 and corresponding pathways against KEGG. **(E)** LncRNA–miRNA–mRNA network predicted by Cytoscape. Target genes of miR-107 are represented as oval-shaped nodes. LncRNAs are represented as triangle-shaped nodes. Red indicates upregulation, and green indicates downregulation. **(F)** Target genes upregulated in DLBCL from GEPIA database (Normal = 337, DLBCL = 47) *p < 0.05.

For the sake of exploring the regulatory mechanisms of miR-107, the target genes of miR-107 were predicted and screened. As shown in the Venn diagram, there was a substantial overlap of 28 target genes detected by 4 prediction algorithms (Figures 5A,B). To estimate the biological implication of miR-107, we then conducted DAVID bioinformatic analysis for functional annotation and possible related pathways. As shown in Figure 5C, GO annotation suggests that miR-107 is mainly related to cell proliferation, cell cycle, and angiogenesis. KEGG annotation indicates that miR-107 is mainly enriched in PI3K-Akt, Oocyte meiosis, Hippo, and AMPK signaling pathways. Furthermore, a representative biomolecular network of approximately 28 target genes and corresponding pathways is shown in Figure 5D. As we can see, nodes of 14-3-3η, AXIN2, CDK6, FGF2, ACTG1, and PPP2R5C unveiled the signaling pathway crosstalk. For example, 14-3-3η was referred to as crosstalk between PI3K-AKT, Oocyte meiosis, vial carcinogenesis, and Hippo signaling pathways. Previous studies report miR-107 as a molecular sponge in signal regulation of cancer (Teng et al., 2015; Lu et al., 2016; Wang P. et al., 2016; Liu et al., 2018; Zhang et al., 2018). Thus, we predicted the lncRNA–miRNA–mRNA ceRNA network and validated the dysregulated lncRNAs in the GSE97336 (*p* < 0.05 and |logFC| ≥ 2). As depicted in Figure 5E, lncRNA CKMT2-AS1 and ZNF767P were upregulated in DLBCL, which might lay the foundation for further study.

To select the appropriate genes in the downstream of miR-107, we detected the expression of these 28 target genes in the GEPIA database and found out that only 6 genes (14-3-3η, CDK6, RRAGC, PP2R5C, OGT, and KIF23) were upregulated in DLBCL (*p* < 0.05, Figure 5F).

### MiR-107 Inhibited DLBCL Tumorigenesis Through Targeting 14-3-3η

**FIGURE 6:**
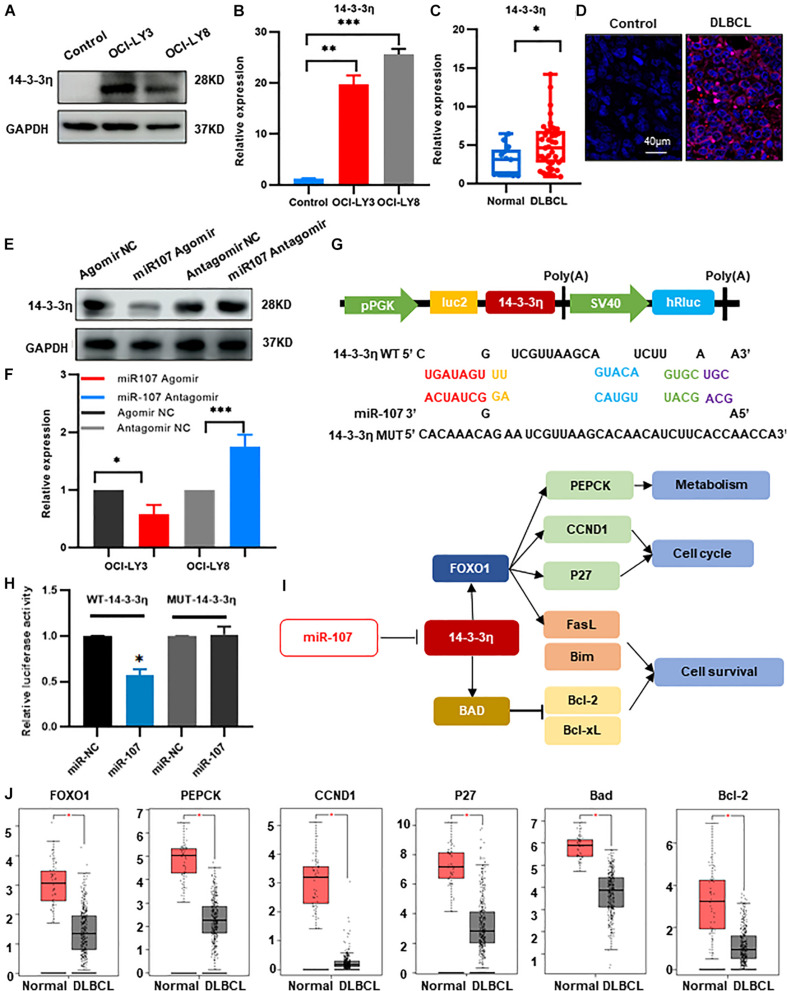
MiR-107 inhibited DLBCL tumorigenesis through targeting 14-3-3η. **(A,B)** Western blot **(A)** and qRT-PCR analysis **(B)** of 14-3-3η expression in DLBCL cell lines. Data were normalized by GAPDH. In the Western blot assay, the ratio of 14-3-3η/GAPDH is shown below the bands. **(C)** QRT-PCR analysis of 14-3-3η in the plasma exosomes of DLBCL patients. **(D)** Analysis at confocal microscopy of 14-3-3η expression in the biopsy tissues of DLBCL patients. **(E,F)** Western bolt **(E)** and qRT-PCR analysis **(F)** of 14-3-3η expression in OCI-LY3 cells transfected with miR-107 Agomir and OCI-LY8 cells transfected with miR-107 Antagomir. **(G)** 14-3-3η binding site of the WT and mutated type with miR-107. **(H)** Co-transfection of HEK293T cells with WT or Mutant (MUT) 14-3-3η 3’-UTR and miR-107 Agomir, as well as miR-107 normal control (miR-107 NC). The relative luciferase activity of HEK293T cells were determined. **(I)** Mechanism diagram of 14-3-3η regulation of FOXO1 and BAD in KEGG. **(J)** Expression of miR-107 target genes in GEPIA database (Normal = 337, DLBCL = 47).

We then confirmed the expression of the above candidate genes by qRT-PCR and found that 14-3-3η was increased in OCI-LY3 and OCI-LY8 cell lines (*p* < 0.05, Figures 6A,B). Furthermore, compared with the healthy controls, the expression of 14-3-3η was also upregulated in the plasma exosomes and tissues of DLBCL patients (Figures 6C,D). Besides, as observed by confocal microcopy, 14-3-3η was mainly stained in the cytoplasm (Figure 6D).

To verify the regulatory role of miR-107 to 14-3-3η, we transfected miR-107 Agomir/Antagomir and scramble controls into OCI-LY3 and OCI-LY8 cell lines, respectively. Expression of 14-3-3η was significantly decreased in the OCI-LY3 cells transfected with miR-107 Agomir and upregulated in OCI-LY8 cells transfected with miR-107 Antagomir (*p* < 0.05, Figures 6E,F). The above results suggest that 14-3-3η expression was upregulated in DLBCL and was negatively regulated by miR-107.

We further applied either wild-type (WT) or mutant 3’-UTR 14-3-3η mRNA containing a luciferase reporter plasmid to detect the direct interaction between miR-107 and 14-3-3η. As shown in Figure 6G, miR-107 contained a complementary binding sequence of 14-3-3η. MiR-107 overexpressed cells transfected with WT 3’-UTR 14-3-3η showed a significant decrease in luciferase activity, and loss of the binding sites eliminated the miR-107 inhibitory effect on luciferase activity (Figure 6H), suggesting the specific binding between miR107 and 3′-UTR in 14-3-3η in DLBCL cells. Figure 6I showed the mechanism diagram of 14-3-3η regulation in the KEGG database. FOXO1, PEPCK, CCND1, P27, BAD, and Bcl-2 were confirmed upregulated in the DLBCL dataset of GEPIA (*p* < 0.05, Figure 6J). Thus, we suggest that miR-107 inhibits DLBCL progression by directly targeting 14-3-3η, leading to inhibition of the downstream oncogenes. These results underline the potential mechanisms of miR-107 in lymphomagenesis and enable the development of novel therapeutic targets for DLBCL management.

## Discussion

Exosomes have been viewed as a major step forward in the liquid biopsies field. Compared with traditional protein-bound miRNA biomarkers, exosome-derived miRNAs were less varied and more informative and extensive in circulation (Van Eijndhoven et al., 2016). Accumulating evidence has suggested exosomal miRNAs could perform as important diagnostic and prognostic biomarkers of cancer (Liu et al., 2020; Tao et al., 2020; Wei S. et al., 2020; Wu G. et al., 2020; Xiao et al., 2020).

To date, a few exosomal non-coding RNAs (ncRNAs) have been described as promising biomarkers or mediators in DLBCL progression (Lopez-Santillan et al., 2018; Feng et al., 2019; Larrabeiti-Etxebarria et al., 2019; Liu and Wang, 2019; Zare et al., 2019a,b). For instance, Zare et al. (2019b) elucidated that exosome-derived miR-155, let-7g, and let-7i acted as non-invasive biomarkers in evaluating therapeutic effects and recurrent risk of DLBCL. Feng et al. (2019) provide exosomal miR-99a-5p and miR-125b-5p as novel diagnostic indicators as well as evaluating prognosis and curative reaction for DLBCL patients. These two miRNAs could also be used as therapeutic targets to potentiate chemosensitivity for the R-CHOP resistance cells. In addition, Van Eijndhoven et al. (2016) observed that the expression of miR-24-3p, miR-127-3p, miR-21-5p, miR-155-5p, and let-7a-5p were upregulated in the plasma extracellular vesicles (EVs) of classical Hodgkin lymphoma (cHL) patients. They declare that serial monitoring of EVs miRNA was suitable for detecting the presence/relapse of individual cHL patients. Hernandez-Walias et al. (2020) provided plasma exosome-derived miR-20a and miR-21 as promising biomarkers for the early detection of cHL in the setting of HIV-1 infection.

In the study, we screened the data from the GEO database and validated two downregulated exosomal miRNAs (miR-107, miR-375-3p) and one upregulated exosomal miR-485-3p in the plasma of DLBCL patients. Consistent with the above studies, the diagnostic performance of miR-107, miR-375-3p, and miR-485-3p in plasma-derived exosomes was inspiring with the AUC of 0.854, 0.769, and 0.739, respectively. Additionally, decreased miR-107 also showed strong prognostic relevance to DLBCL patients by indicating advanced Ann Arbor stage, ECOG, LDH, β_2_-MG, and IPI score in our results. This gave us a hint that miR-107 may participate in the tumorigenesis and progression of DLBCL. Collectively, the above results identified an unprecedented diagnostic significance for circulating exosomal miRNAs in patients with DLBCL, which requires further validation in a larger cohort. In addition, the association between exosomal miRNAs and outcomes in newly diagnosed DLBCL patients is worth exploring in future studies.

MiR-107 has been reported as a biomarker and modulator among several cancer types, i.e., non-small cell lung cancer (NSCLC), breast cancer, gastric cancer, colorectal cancer, and cervical cancer. Except for the peripheral blood, miR-107 was also detected in the urine EVs of prostate cancer patients. A combination with miR-31-5p was revealed as a novel diagnostic biomarker (Lekchnov et al., 2018). Based on the current knowledge (Table 1), most of the studies support miR-107 as a tumor suppressor in tumorigenesis by attenuating cell survival and metastasis as well as potentiating chemosensitivity. Intriguingly, some other studies declare a tumor promoting function. Ren et al. (2019) suggest that miR-107 could promote tumor progression in an exosome delivery way. They prove that exosomes generated by gastric cancer cells delivered miR-107 to myeloid-derived suppressor cells result in cell proliferation and chemotherapy resistance. This diversity of opinion discrepancy may be due to the tumor types and origin of miRNAs, which need more studies to clarify.

**TABLE 1:** MiR-107 performed as a diagnostic, prognostic indicator, and participated in cancer progression.

Cancer type	Expression	Functions and mechanisms
Breast cancer	↓	Inhibits cell proliferation, cell-cycle and invasion through BDNF (Ai et al., 2018)
	↓	Inhibits cell proliferation, colony formation, migration and invasion and promotes apoptosis through targeting SIAH1 (Zhang et al., 2016)
	↓	Relates with histological grade index of ER-positive breast cancer (Tsunoda et al., 2018)
	↑	Performs predictive role in the triple-negative breast cancer (Hong et al., 2020; Terkelsen et al., 2020)
Gastric cancer	↑	Associates with tumor progression characteristics and acts as an independent prognostic factor for OS and DFS (Inoue et al., 2012)
	↑	Promotes cell growth, migration, and invasion by targeting NF1 (Wang S. et al., 2016)
Lung cancer	↓	Inhibits cell proliferation, migration and arrest cell cycle by targeting TGFβR2 (Wu Z. et al., 2020)
	↓	Inhibits paclitaxel resistance, metastasis, proliferation and survival through Bcl-w/PI3K-AKT (Lu et al., 2017)
	↓	Inhibits cancer progression by targeting EGFR (Wang et al., 2017)
	↓	Correlates with tumor progression characteristics and inhibits cancer growth by targeting BDNF and PI3K/AKT pathway (Xia et al., 2016)
Hepatocellular carcinoma	↑	Promotes tumor progression through miR-107/CPEB3/EGFR axis (Zou et al., 2016)
Renal cell carcinoma	↓	Inhibits cell proliferation and invasion by targeting EIF5 (Song et al., 2015)
Meningioma	↓	Correlates with the increasing histopathological grade (Katar et al., 2017)
Prostate cancer		MiR-107-miR-26b-5p predict prostate cancer with AUC = 0.93 and *p* = 0.0012 (Lekchnov et al., 2018)
Glioblastomas	↓	MiR-107-miR-331 associate with poorer prognosis (*p* = 0.033) (Hermansen et al., 2017)
Colorectal cancer	↑	Induces chemoresistance through CAB39-AMPK-mTOR pathway (Liang et al., 2020) HOTAIRM1/miR-107/TDG axis regulates cell proliferation, invasion, and migration (Li et al., 2020)

*↑, Upregulation; ↓, Downregulation.*

Regarding miR-375, Su et al. (2019) provide that it was significantly upregulated in the serum exosomes of ovarian cancer. The overexpressed miR-375 showed independent diagnostic power and enhanced the diagnostic accuracy of ovarian cancer. In addition, Huang et al. (2015) suggest that plasma exosomal miR-375 is a promising prognostic biomarker for castration-resistant prostate cancer patients. In the case of miR-485, it was proved as a non-invasive biomarker for identifying high-risk thyroid nodules and monitoring the therapeutic reaction of NSCLC. Besides, downregulating miR-485 expression could abrogate cell proliferation and survival and enhance radiation sensitivity in NSCLC (Ma et al., 2016; Pilli et al., 2017).

In our results, after upregulating miR-107 expression with miR-107 Agomir, we observed an abrogation of tumor progression and metastasis, and downregulating miR-107 expression showed the opposite effects. Nevertheless, the biological mechanism underlying this effect remains unclear in DLBCL.

Building from previous studies, miR-107 could suppress cancer progression in many ways, such as targeting oncogene eukaryotic translation initiation factors (eIFs); transforming growth factor beta receptor 2 (TGFβR2); epidermal growth factor receptor (EGFR); or attenuating AMPK-mTOR, NOTH2, and other pathways (Table 1). In our result, we identified interactions between miR-107 and 14-3-3η by dual-luciferase assay.

Scaffold protein 14-3-3 containing seven isoforms (β, γ, ξ, σ, ε, τ, η) is reported to modulate cell proliferation, cell cycle, apoptosis, angiogenesis, and DNA repair (Pennington et al., 2018). 14-3-3 has been proved to influence biological processes by binding and influencing protein structure, activity, or stability, promoting protein complex formation, and altering protein subcellular localization. Although knocking down of 14-3-3η disrupts the 14-3-3-ligand association. In DLBCL, upregulated 14-3-3 has been proved to play a stimulatory role in DLBCL progression. For instance, upregulated 14-3-3ξ was reported to promote chemoresistance of DLBCL cells to CHOP therapy (Maxwell et al., 2009). High expression levels of 14-3-3ε is an independent predictor of chemotherapy resistance and poor prognosis for advanced extranodal natural killer/T cell lymphoma (ENKTL) patients (Qiu et al., 2017). 14-3-3η is reported to mediate cell proliferation, cell apoptosis, and cell cycle by combining with FOXO1, FOXO3a, BAX, BAD, and HIF-1α (Trinh et al., 2013; Qiu et al., 2019). According to the above, we suspect that miR-107 might abrogate DLBCL progression through downregulating 14-3-3η.

Circulating exosomal miRNAs (miR-107, miR-375-3p, and miR-485-3p) as promising diagnostic indicators have opened up new avenues for developing non-invasive methods in the diagnosis or monitoring of DLBCL. We suggest that dysregulated miR-107 might be the Achilles heel in the DLBCL progression, which would enrich our therapeutic armamentarium against DLBCL.

However, to improve the clinical value of exosomal miRNAs, there are still some areas to be improved. We should not only enlarge the sample size but also include therapy strategy, therapeutic response, and prognosis in future observations. In addition, protein aggregates and lipoprotein contaminants are also problems limiting the development of liquid biopsy technology. Hence, great efforts have to be made for the clinical application of exosomes.

## Data Availability Statement

The original contributions presented in the study are included in the article/supplementary material, further inquiries can be directed to the corresponding author/s.

## Ethics Statement

The studies involving human participants were reviewed and approved by the Shandong Provincial Hospital. The patients/participants provided their written informed consent to participate in this study. The animal study was reviewed and approved by Animal Care and Use Committee of Shandong Provincial Hospital. Written informed consent was obtained from the individual(s) for the publication of any potentially identifiable images or data included in this article.

## Author Contributions

JL designed, performed the experiment, and wrote the manuscript. SH and YH subscribed to experimental resources. JY, SR, YZ, and YC advised on experimental designs and methodology. XZ performed supervision and reviewed the manuscript. XW supervised the experiment and provided funding. All authors contributed to the article and approved the submitted version.

## Conflict of Interest

The authors declare that the research was conducted in the absence of any commercial or financial relationships that could be construed as a potential conflict of interest.

## Footnotes

1 https://www.ncbi.nlm.nih.gov/geo/

2 http://www.targetscan.org/vert_71/

3 http://www.mirdb.org

4 http://mirtarbase.mbc.nctu.edu.tw/php/index.php

5 https://pictar.mdc-berlin.de/cgi-bin/PicTar_vertebrate.cgi

6 http://zmf.umm.uni-heidelberg.de/apps/zmf/mirwalk2/index.html

7 https://david.ncifcrf.gov
